# Cellular tropism of SARS-CoV-2 in the respiratory tract of Syrian hamsters and B6.Cg-Tg(K18-ACE2)2Prlmn/J transgenic mice

**DOI:** 10.1177/03009858211043084

**Published:** 2021-09-01

**Authors:** Hui-Ling Yen, Sophie Valkenburg, Sin Fun Sia, Ka Tim Choy, J. S. Malik Peiris, Karen H. M. Wong, Nicholas Crossland, Florian Douam, John M. Nicholls

**Affiliations:** 1 The University of Hong Kong, Pok Fu Lam, Hong Kong; 2 Boston University, Boston, MA, USA

**Keywords:** COVID-19, SARS-CoV-2, severe acute respiratory syndrome, coronavirus, pathology, immunohistochemistry, ultrastructure

## Abstract

Several animal models have been developed to study the pathophysiology of SARS-CoV-2 infection and to evaluate vaccines and therapeutic agents for this emerging disease. Similar to infection with SARS-CoV-1, infection of Syrian hamsters with SARS-CoV-2 results in moderate respiratory disease involving the airways and lung parenchyma but does not lead to increased mortality. Using a combination of immunohistochemistry and transmission electron microscopy, we showed that the epithelium of the conducting airways of hamsters was the primary target for viral infection within the first 5 days of infection, with little evidence of productive infection of pneumocytes. At 6 days postinfection, antigen was cleared but parenchymal damage persisted, and the major pathological changes resolved by day 14. These findings are similar to those previously reported for hamsters with SARS-CoV-1 infection. In contrast, infection of K18-hACE2 transgenic mice resulted in pneumocyte damage, with viral particles and replication complexes in both type I and type II pneumocytes together with the presence of convoluted or cubic membranes; however, there was no evidence of virus replication in the conducting airways. The Syrian hamster is a useful model for the study of SARS-CoV-2 transmission and vaccination strategies, whereas infection of the K18-hCE2 transgenic mouse results in lethal disease with fatal neuroinvasion but with sparing of conducting airways.

Since the emergence of a novel coronavirus (SARS-CoV-2) in China with rapid spread around the world, there has been a concentrated effort to understand the pathophysiology of COVID-19, and to compare it with the lesions caused by other coronaviruses such as SARS-CoV-1 and MERS-CoV. One of the main focal points of research in the COVID-19 outbreak has been to find a laboratory animal model that would faithfully reproduce a similar temporal pattern of pulmonary changes as in the human infection. The initial postmortem studies on human patients with fatal COVID-19 appeared to show a histological picture similar to that seen with SARS-CoV-1 infection (reviewed in Calabrese et al^7^); however, in retrospect, this may have represented a publication bias because, similar to the 2003 outbreak, there were few studies done on tissues from the conducting airways or from patients in the early stages of disease. Thus, it was challenging to determine whether the patterns reported were those caused by the virus, or secondary to medical treatment in intensive care. There was little information on mild to moderate disease, and the published ex vivo studies were limited to the first 72 hours of infection, as this was the extent that the cultures could be successfully maintained.^18^ The human postmortem studies showed 3 overlapping phases of pulmonary disease: an initial exudative phase in which the walls of the alveoli had hyaline membranes together with the presence of pulmonary edema, a second phase with proliferation of type II pneumocytes and often with formation of syncytia, and a final organizing phase with fibrosis, proliferation of vessels, and squamous metaplasia in the conducting airways.^31^ Immunohistochemistry in SARS-CoV-1 and SARS-CoV-2 patients showed immunolabeling for virus in epithelial cells, endothelial cells, and exudate in the first 2 phases, but not in the third.^30^ There has been conflicting data on virus presence by ultrastructure.^47^ As the 2 most common laboratory animals for study of SARS-CoV-2 pathology have been the Syrian hamster and the B6.Cg-Tg(K18-ACE2)2Prlmn/J transgenic mouse, the objective of this study was to determine which cells in the respiratory tract are infected with SARS-CoV-2, to characterize the relative benefits and drawbacks of these animal models.

## Methods

### Infection

In Hong Kong, male golden Syrian hamsters (*N* = 9), 4 to 8 weeks old, were obtained from the Laboratory Animal Services Centre, Chinese University of Hong Kong. The animal ethics was approved by the Committee on the Use of Live Animals in Teaching and Research at the University of Hong Kong (CULATR# 5323-20). The hamsters were originally imported from Harlan (Envigo, UK) in 1998. They were infected intranasally with 8 × 10^4^ TCID_50_ in 80 µL phosphate-buffered saline (PBS) of SARS-CoV-2 (BetaCoV/Hong Kong/VM20001061/2020 virus; GISAID# EPI_ISL_412028), which was isolated in Vero E6 cells from the nasopharynx aspirate and throat swab of the first confirmed COVID-19 patient in Hong Kong. On days 2, 5, and 7 post-inoculation, nasal turbinate, lungs, heart, duodenum, liver, spleen, and kidney were collected to monitor viral replication and histopathological changes. The animals used were part of a study on the role of transmission^46^ and monoclonal antibody therapy, so insufflation of the lungs before sampling was not possible. Respiratory tract tissues were fixed in 10% formalin at room temperature for a minimum of 24 hours. They were then dehydrated by immersion in graded ethanol, cleared in xylene, and processed into paraffin blocks. The paraffin-embedded tissue samples were then cut into 5-µm-thick sections with a microtome and mounted onto coated slides and dried overnight.

In Hong Kong, K18 hACE2 heterozygous female mice (B6.Cg-Tg(K18-ACE2)2Prlmn/J; 14–20 weeks old; *n* = 8) were infected with 10^4^ TCID_50_ in 25 µl PBS of SARS-CoV-2 (βCoV/Hong Kong/VM20001061/2020 strain [GISAID ID: EPI_ISL_412028]). All experimental procedures were conducted in accordance with the standards and approved by the Committee on the Use of Live Animals in Teaching and Research (approval #5511-20), The University of Hong Kong. Mice were euthanized by intraperitoneal injection of pentobarbital at days 2, 3, and 4 post-infection. The lung was perfused with 1 mL of 10% neutral-buffered formalin via the trachea, dissected, and washed in PBS, and stored in 10% neutral buffered formalin. For the k18-hACE2 time course, images of hematoxylin and eosin–stained sections and immunohistochemistry-labeled section were obtained from Boston University’s National Emerging Infectious Diseases Laboratories (NEIDL) as part of a study on viral neuroinvasion.^8^ No animals were excluded from data analyses. As this was a descriptive study, no estimation of sample size or statistical analysis was performed.

### Immunohistochemistry

#### SARS-CoV Nucleoprotein

Immunohistochemistry on formalin-fixed and resin-embedded tissues was performed using a rabbit polyclonal antibody (Sinobiological 40143-T62) against SARS-CoV nucleoprotein, as detailed in a previous publication.^46^ For semi-thin immunohistochemistry, sections were etched with saturated NaOH, blocked with H_2_O_2_, and incubated with the previously mentioned antibody at a dilution of 1/100, followed by Impress anti-Rabbit-HRP (Vector Labs), developed with NovaRed (Vector Labs) and counterstained with toluidine blue.

#### SARS-CoV-2 Spike Protein

For formalin-fixed and paraffin-embedded tissues monoplex DAB immunohistochemistry was conducted using a Chromomap DAB IHC kit (Roche) with CC1 antigen retrieval at 95 °C for 32 minutes. Primary antibody incubation was conducted with a mouse mAB specific to SARS-CoV-1/2 Spike Protein (Cell Signaling Technology, 2B3E5), followed with a rabbit anti-mouse linking antibody IgG1+IgG2a+IgG3 antibody (Abcam, ab133469) at 37 °C for 20 minutes (1:1,000), and finally with a pre-dilute ImmPRESS polymer goat anti-rabbit HRP conjugated secondary (Vector Labs MP-7451-50)  for 20 min at 37 °C. Immunoreactivity was developed via DAB followed by nuclear counterstaining with hematoxylin and bluing reagents (Roche).

### Transmission Electron Microscopy (TEM)

Hamster and mouse lung samples (fixed in 10% neutral buffered formalin) were processed directly into resin for electron microscopy rather than paraffin. After chemical etching, immunohistochemistry for CoV-nucleoprotein was performed on the resin blocks, which allowed a correlation of the immunohistochemistry with the ultrastructural identification of foci of viral replication. We used hamster tissues which were fixed after 2, 4, and 5 days after infection. Because there is a sampling challenge in finding immunoreactive areas, tissues were embedded into multiple 5 mm blocks of resin, and then trimmed down to the conventional 2 mm blocks for ultrastructural analysis once immunoreactive foci were identified. We used previously published criteria for the ultrastructural morphology of the hamster respiratory tract.^5^ Additional electron microscopic images of the lung of 12- to 14-week-old K18-hACE2 mice at day 6 after SARS-CoV-2 infection (intranasal inoculation with 10^6^ PFU of a 2019-nCoV/USA-WA1/2020 isolate obtained from BEI resources; NCBI accession number: MN985325) were obtained from Boston University’s National Emerging Infectious Diseases Laboratories (NEIDL) as part of a study on viral neuroinvasion.^8^ Immunogold labelling was attempted using Electron Microscopy Solution 25544 EM-kit GAM-6/GAR-10/BSA Goat-anti-Mouse IgG 6 nm, and Goat-anti-Rabbit 10 nm using manufacturer’s protocol, and rabbit polyclonal antibody (Sinobiological 40143-T62) for SARS-CoV nucleoprotein.

## Results

### Day 2 Post-Infection

**Figures 1–6.**
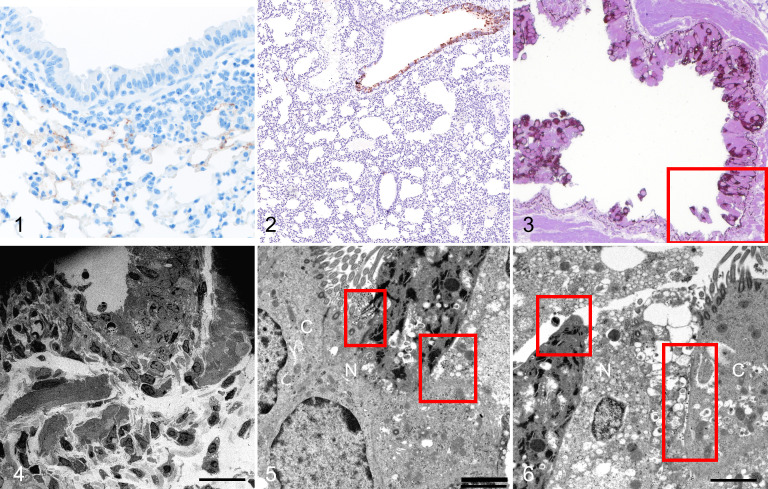
SARS2-CoV-2 infection, lung, K18-hACE2 mouse and hamster, day 2 post-infection. **Figure 1**. K18-hACE2 mouse. There is minimal interstitial mononuclear cell infiltrate with scant immunolabeling for SARS-CoV-2 spike antigen mainly in alveolar type I pneumocytes adjacent to areas of inflammation. **Figure 2.** Hamster. There is immunolabeling for SARS-CoV nucleoprotein in the bronchiolar but not in the alveolar epithelium. **Figure 3**. Hamster. The bronchiolar epithelium is denuded or multilayered. Immunolabeling for SARS-CoV nucleoprotein is present along the basement membrane. Resin-embedded semi-thin section. **Figure 4**. Bronchiolar epithelium, hamster. Transmission electron microscopy (TEM). Ultrastructure of the boxed area in Figure 3, with denudation of the epithelium. Bar: 20 µm. **Figure 5**. Bronchiolar epithelium, hamster. TEM. There is cytoplasmic swelling of a non-ciliated bronchiolar epithelial cell (N). Large number of viral particles (red boxes) are seen at the surfaces of the ciliated cell (C) and non-ciliated cell (N). Bar: 2 µm. **Figure 6**. Bronchiolar epithelium, hamster. TEM. There is marked cytoplasmic swelling and vacuolation of a non-ciliated cell (N). Aggregates of viral particles are present in secretory vesicles, on the surface, and in the intercellular space between the ciliated cell (C) and the non-ciliated cells (N) (boxes). In contrast to the non-ciliated cell, the cell membrane of the ciliated cell is relatively intact. Bar: 2 µm.

Immunohistochemistry scoring data are shown in Table 1. In the K18-hACE2 mouse lung (Fig. 1), there was minimal interstitial mononuclear infiltrate. There was low immunolabeling for SARS-CoV-2 spike antigen mainly in alveolar type I pneumocytes neighboring the areas of inflammation. There was no immunolabeling of the bronchioles identified.

**Table 1.** Immunohistochemistry scores for viral nucleoprotein in Syrian hamsters and K18-hACE2 mice infected with SARS-CoV-2.

DPI	Hamster			K18-hACE2		
	Nasal	Bronchiole	Pneumocyte	Nasal	Bronchiole	Pneumocyte
2	3^a^	3	0	1–2	0	1–2
4	2	2	1–2	0–1	0	1–3
6–8	0	0	1	0	0	2
14	0	0	0	0	0	0

Abbreviation: DPI, days post-infection.

^a^0, 0 SARS-CoV immunoreactive cells; 1, 0% to 5% immunoreactive cells at 400× magnification; 2, 5% to 25% immunoreactive cells at 400× magnification; 3, >25% immunoreactive cells at 400× magnification.

The hamster tissues showed no significant inflammatory cells within the bronchiolar lumens or in alveoli. By immunohistochemistry, antigen was confined to the epithelial cells of the bronchioles (Fig. 2). Semi-thin immunohistochemistry performed on the resin-embedded blocks, and nucleoprotein antigen was present in the respiratory bronchioles but not the alveoli. This was associated with marked denudation of epithelial cells, and aggregates of necrotic cells were identified within the bronchial lumens (Fig. 3). There was staining of the basement membrane in the denuded area in the immunohistochemistry preparations; however, no viral particles were detected in this area by electron microscopy (Fig. 4). This may represent either nonspecific staining or binding of viral nucleoprotein to extracellular matrix.^49^ Ultrastructural examination showed the presence of viral particles in membrane bound vesicles within both the ciliated (Fig. 5) and non-ciliated cells. However, the non-ciliated epithelium showed far more cellular damage, with cytoplasmic vacuolation and intracellular swelling, compared to the ciliated epithelium (Fig. 6; Suppl. Figs. S1, S2). The non-ciliated cells in the infected animals also showed cellular damage even when there was no evidence of viral infection by immunohistochemistry or electron microscopy.

### Day 4 Post-Infection

**Figures 7–12.**
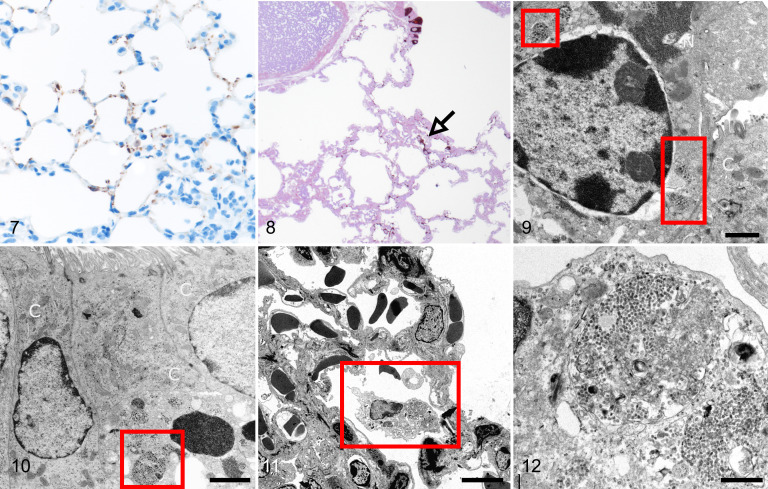
SARS2-CoV-2 infection, lung, K18-hACE2 mouse and hamster, day 4 post-infection. **Figure 7**. K18-hACE2 mouse. There is an interstitial and perivascular infiltrate of mononuclear cells, and greater immunolabeling for SARS-CoV-2 spike protein than at day 2   within alveolar type I and type II pneumocytes. **Figure 8**. Hamster. There is immunolabeling for SARS-CoV nucleoprotein in bronchiolar epithelial cells, focally along alveolar septa, and in an alveolar macrophage (arrow). Resin embedded semi-thin section. **Figure 9**. Bronchiolar epithelium, hamster. Transmission electron microscopy (TEM). A non-ciliated cell (N) contains large aggregates of viral particles (boxes). Bar: 2 µm. **Figure 10**. Bronchiolar epithelium, hamster. TEM. A ciliated epithelial cell (C) contains large membrane-bound aggregates of viral particles (box) on the basal aspect of the cell. Bar: 2 µm. **Figures 11, 12**. Alveolus, hamster. TEM. The immunoreactive alveolar macrophage identified in Figure 8 contains large numbers of membrane-bound viral particles. The adjacent epithelial cells show no swelling of the cytoplasm. Bars: 5 µm (Fig. 11); 500 nm (Fig. 12).

The K18-hACE2 mouse lung had increased numbers of mononuclear inflammatory cells in the interstitium and around blood vessels. The SARS-CoV-2 spike antigen was detected in neighboring alveolar type I and type II pneumocytes (Fig. 7). No staining of the bronchioles was detected.

In the hamsters, immunoreactive cells were present in both non-ciliated as well as ciliated cells in the bronchioles, and linear immunolabeling extended into the alveolar walls (Fig. 8). There was also positive immunolabeling of an alveolar macrophage identified (Fig. 8, arrow). Ultrastructurally, large membrane-bound aggregates of viral particles were identified in the cytoplasm of non-ciliated (Fig. 9, Suppl. Fig. S3) and ciliated (Fig. 10, Suppl. Fig. S4) cells. The alveolar macrophage contained membrane-bound viral particles (Figs. 11, 12); however, careful examination of the adjacent pneumocytes failed to demonstrate cell swelling or intracellular viral particles. No significant endothelial swelling, cell death, or thrombosis was identified and there was no immunolabeling or ultrastructural evidence of virus in the endothelial cells.

### Day 5 Post-Infection

**Figures 13–20.**
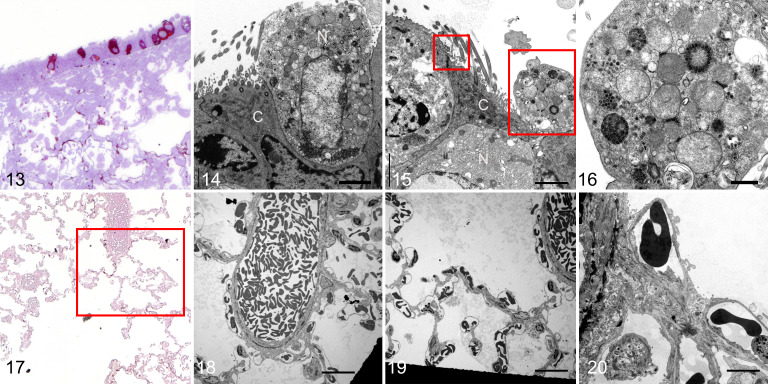
SARS2-CoV-2 infection, lung, hamster, day 5 post-infection. **Figure 13.** Immunolabeling for SARS-CoV nucleoprotein in bronchiolar epithelial cells and focally along alveolar septa. Resin-embedded semi-thin section. **Figure 14.** Bronchiolar epithelium. Transmission electron microscopy (TEM). A non-ciliated cell (N) has cell swelling, membrane fragmentation, and cytoplasmic membrane-bound aggregates of large numbers of viral particles. In contrast, the adjacent ciliated cell (C) shows minimal cytopathic changes. Bar: 2 µm. **Figure 15**. Bronchiolar epithelium. TEM. A ciliated cell (C) has small clusters of extracellular viral particles on the cell surface (small box). Large box: a fragment of a non-ciliated cell (N). Bar: 2 µm. **Figure 16.** Bronchiolar epithelium. TEM. The non-ciliated cell fragment in Figure 15 contains large numbers of membrane- and non-membrane-bound viral particles, including one aggregate of closely packed particles. Bar: 500 nm. **Figure 17.** Immunolabeling for SARS-CoV nucleoprotein along alveolar septa. Resin-embedded semi-thin section. **Figures 18–20.** Alveoli. TEM. The boxed area in Figure 17 have normal alveolar walls with no swelling. No intracellular or extracellular virus is evident. The black areas in Figures 18 and 19 are the meshes of the grids. Bars: 20 µm (Figs. 18, 19); 5 µm (Fig. 20).

The hamster lung had immunoreactive ciliated and non-ciliated bronchiolar epithelial cells and in the adjacent alveolar walls, but there was no associated denudation of bronchiolar or alveolar epithelial cells (Fig. 13). Ultrastructural examination showed marked cellular swelling and membrane fragmentation of the non-ciliated bronchiolar epithelial cells associated with viral replication complexes (Fig. 14). The adjacent ciliated cells had preservation of the cilia structure and viral aggregates on the cell surface (Fig. 15 [small box]; Suppl. Figs. S5, S6). One partially denuded non-ciliated cell (Fig. 15 [large box]) had large compact aggregates of viral particles that appeared to be devoid of a double-wall membrane (Fig. 16). This is a feature that has not been readily identified in Vero cell cultures.^12^ In the immunoreactive alveolar region (Fig. 17), there was no ultrastructural evidence of virus within pneumocytes or endothelial cells or of endothelial or epithelial damage (Figs. 18–20), and the bronchiolar basement membrane was also non-reactive to viral nucleoprotein.

**Figures 21–24.**
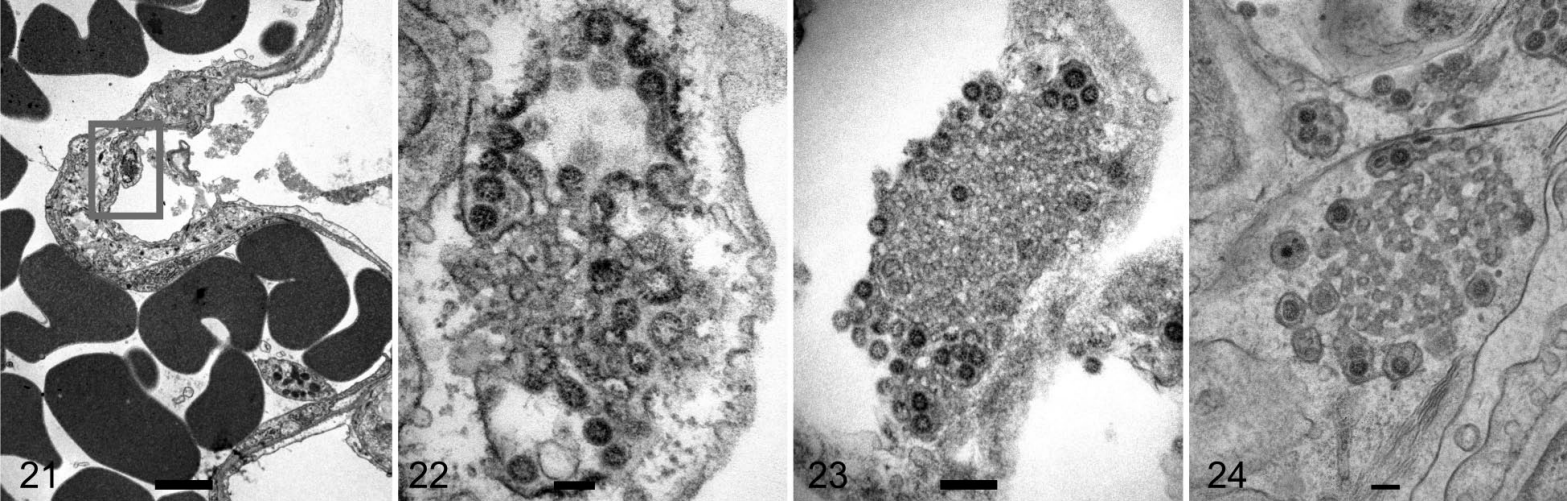
SARS2-CoV-2 infection, lung, K18-ACE2 transgenic mouse. Transmission electron microscopy. **Figures 21, 22.** Day 4 post-infection. A type I pneumocyte has swollen cytoplasm (box) with a cubic membrane of viral particles budding from the rim and a central convoluted aggregate of membrane material (Fig. 22). Bar: 2 µm (Fig. 21); 100 nm (Fig. 22). **Figure 23.** Day 4 post-infection. A cubic membrane with a central convoluted aggregate of material and a peripheral rim of budding virus. Bar: 200 nm. **Figure 24.** Day 6 post-infection. A pneumocyte contains a central convoluted membrane and a peripheral rim of membrane-bound virus. Bar: 500 nm.

Ultrastructural examination of the lungs of K18-hACE2 mice at days 3, 4, and 6 post-infection showed more severe damage to type I pneumocytes compared to that in infected hamsters. Specifically, there was ballooning of the cytoplasm, secretory vesicles containing viral particles, and aggregates of membranous material surrounded by viral particles consistent with convoluted or cubic membranes (Figs. 21–24).

Despite multiple etching and adhesion techniques, there was no consistent immunogold localization of anti-nucleoprotein antibody to viral aggregates using immunoelectron microscopy, even though the immunohistochemistry using semi-thin resin-embedded samples identified immunoreactive areas that correlated with the labeling in formalin-fixed tissues.

## Discussion

In Syrian hamsters infected with SARS-CoV-2 that were part of a study on virus transmission, we found that that replication began in the conducting airways at day 2 and subsequently involved the parenchyma. Although maximal infectious viral titers peaked on days 2 to 3 after infection, antigen was maximally detected by immunohistochemistry on day 5. After day 5 there was resolution of viral load and clinical signs, with most animals recovering by day 14.^46^ By immunohistochemistry, antigen was widespread in the lungs on day 5 of the infection, but none was identified on day 7. We found 2 puzzling observations from evaluation of the lung lesions at this early stage of disease. First, there was a discordance between the distribution of antigen and the level of lung damage. In particular, there were areas of antigen without lung lesions, and conversely areas of parenchymal consolidation without detectable antigen. This feature was reported previously in hamsters infected with SARS-CoV.^37^ Second, as we found a rapid clearance of viral antigen between days 5 and 7 after infection,^46^ we compared of the presence of antigen based on immunohistochemistry with the detection of viral particles within the bronchioles and alveoli by electron microscopy, as the latter is the definitive method for identification of actual virus. Pneumocytes in infected hamsters had immunoreactive cells but no evidence of viral particles after day 5 post-infection, whereas in the K18-hACE2 mice there was both immunoreactivity and ultrastructural evidence of infection of type I and type II pneumocytes at day 6, but no infection of the conducting airways.

Infection of the K18-hACE2 transgenic mice showed more evidence of pneumocyte damage together with cytoplasmic viral aggregates. Thus, this mouse model may be better than the hamster model for understanding parenchymal lung disease. However, the profound neuro-invasion at day 7 contributing to death in the K18-hACE2 model remains a limitation of this model and also means that long-term studies will be challenging.^8^

With regard to the cellular tropism in the 2 animal models examined, our findings showed that in the hamster, the conducting airway was the main target, with both ciliated and non-ciliated cells showing intracellular virus. Non-ciliated cells had more severe damage than ciliated cells, characterized by cytoplasmic swelling, loss of membrane integrity, blebbing, and fragmentation, but this was associated with more abundant virus production in non-ciliated than in ciliated cells. This difference in virus production and cellular response can be attributed to the biological function of the 2 types of cells, as ciliated cells are more specialized and are not secretory, unlike the non-ciliated club cell that has more Golgi apparatus, secretory granules, and endoplasmic reticulum. Ultrastructure of the normal conducting airway of the hamster has shown that it is not identical to the human, in that there are no goblet cells, the epithelium is not as pseudostratified, and there appears to be an equal distribution of ciliated and non-ciliated cells.^5^ The bronchiolar changes seen at day 2 of infection in the hamster were similar to that reported in other viral infections, namely, multilayering of the epithelium and cellular swelling, similar to those reported in other studies.^15^ Pneumocytes did not appear to be a target for SARS-CoV-2 in the hamster even though they can express ACE2. In the K18-hACE2 mouse, infection was limited to the alveolar type I and type II pneumocytes; although the conducting airways expressed ACE2, there was no evidence of virus infection at the immunohistochemical or ultrastructural level. We have not been able to determine the reason for this lack of viral replication in bronchiolar epithelium. Furthermore, alveolar macrophages in the hamsters contained aggregates of viral particles, but this was not present in mouse macrophages.

Though immunoelectron microscopy was not successful on the hamster tissues, the viral antigen present on the surface of pneumocytes was considered to probably represent surface deposition from proximal (bronchiolar) infected epithelial cells, or uptake by pinocytic vesicles. The cubic or convoluted membranes identified in the mouse pneumocytes were not present in the respiratory epithelium of hamsters, and though mentioned in cell cultures^12^ do not appear to have been described in animal models of coronavirus infection before.

The type I pneumocyte is not able to replicate, and compared to the type II pneumocyte has less subcellular machinery needed for protein production such as Golgi and endoplasmic reticulum. It is possible that the cubic membranes seen in the pneumocytes and not the bronchi of mice represent an abortive stage of virus replication and release.

In the hamster, where ACE2 is not present on alveolar type I pneumocytes, immunolabeling for viral antigen of the basement membrane at day 2 post-infection, plus the loss of antigen in non-inflamed tissues from days 5 to 7, suggests clearance of surface antigen rather than immune-mediated destruction of infected cells. At later stages of the disease, hamsters had histologic evidence of pneumocyte damage, corresponding to type II pneumocyte hyperplasia and endothelial swelling, but there was no evidence of direct viral infection based on immunohistochemistry and electron microscopy. This may be explained by recent publications showing that SARS-CoV-2 infection of airway epithelial cells can lead to production of pro-inflammatory cytokines and interferons mediators, and these can damage pneumocyte cell lines and endothelial cells in a paracrine fashion without direct viral infection.^11,50^ A recent ultrastructural examination of the endothelium of the hamster lungs supports this hypothesis as it showed there was damage to the endothelium without the presence of virus.^1^

Even though the alveolar structure is similar between the hamster and the human lung, there is a marked difference in the distal conducting airway anatomy of the hamster compared to that of humans. Specifically, humans have a distinct distal airway that includes respiratory bronchioles.

The time course of infection that we have seen in hamsters infected with SARS-CoV-2 is similar to other respiratory viruses. Studies with human parainfluenza virus performed in 1964 showed desquamation of cells in the lumens of the bronchi on day 3, peribronchial inflammation on day 5, and infrequent involvement of the alveoli.^6^ It was reported that the bronchial epithelium had many cells that were thickened and distorted into folds. By day 13 there was little evidence of residual tissue damage and a normal ciliated epithelium was restored. Infection with human metapneumovirus^26^ also showed mild pulmonary disease and it was reported that the hamsters cleared virus by day 6; there were no pathological changes reported in these animals. The conclusion from these studies was that hamsters would be useful and helpful for vaccine studies but not necessarily from understanding pathogenesis. After the SARS 2003 outbreak, studies in hamsters identified that the lethality depended on the viral strain: though no hamsters died when infected with the Urbani strain, 3 out of 20 hamsters died when challenged with the Frk-1 strain, which differed from the Urbani strain by the L1148F mutation in the S2 domain.^38,39^

Several published reviews on animal models for the study of SARS-CoV-2 infection have concluded that hamsters are the best model and resemble mild to moderate COVID in humans. Infected hamsters had a high viral load in the upper respiratory tract but also developed more lower respiratory disease than standard laboratory mice, and they support efficient transmission of SARS-CoV-2, are easier to handle than ferrets (which do not develop moderate respiratory disease and less susceptible in transmission studies), and are considerably less expensive with fewer ethical concerns than nonhuman primates.^9,10,13,14,17,20,28,33,40,43,53^ Recent publications used hamster models for SARS-CoV-2 vaccine development, and these have included the use of a number of backbones or vectors, including rabies,^22^ measles,^25^ Newcastle disease,^48^ yellow fever,^42^ monoclonal antibodies,^2,23,24,44^ and nanobodies.^29,51^ The hamster model has also been used to study new therapeutic agents for the prevention and treatment of infection including favipiravir,^21^ hydroxychloroquine,^41^ methylprednisone,^52^ plant extracts,^34^ interferon,^16^ remdesivir,^52^ anti-leprosy medications,^54^ and ranitidiine bismuth citrate.

With viral infections other than coronavirus, most studies have found that mice are superior to hamsters for studying viral lung injury, and distal airway epithelial cells can be a source of regenerating pneumocytes derived from cytokeratin 5-immunoreactive stem cells forming structures called nascent pods.^35^ As the distal airways of mice and humans have different structures, it has been postulated that this may lead to a different response in mice than in humans.^4,27^ There has been limited study on the role of non-ciliated cells contributing to lung damage in hamsters.^36^

Our studies of K18-hACE2 mice and hamsters used young rather than aged animals. After the 2003 SARS pandemic, studies of SARS-CoV-1 pathogenesis found that even though mice had little evidence of clinical disease after infection, aged mice had greater disease severity than younger mice.^3^ Most published studies on infection with SARS-CoV-2 have used hamsters from 4 to 6 weeks of age, as these are more readily available than aged hamsters. Three studies^19,32,45^ compared disease severity in aged and young hamsters. In 2 studies, the same virus inoculation titer and route of administration were used. The first^32^ showed that the age groups had no difference in mortality, and replication in the upper respiratory tract was the same as the lower respiratory tract, but younger hamsters had an earlier and stronger immune response. In both age groups, necrotic endothelial cells were identified, separated from the basement membrane by subendothelial inflammatory cells. In contrast, a second study^45^ using aged hamsters (10–20 months) found more severe disease and greater mortality, and one animal developed myocardial disease and thrombus formation in the left atrium. A third study^19^ used 1-month-old and 7- to 8-month-old hamsters and found no appreciable difference in viral titer in the respiratory tissues on days 3 and 6 after infection but apparent differences in weight loss and the timeline for disease progression/recovery.

As a result of our ultrastructural and immunohistochemical investigations, there are a number of conclusions which can be made about the usefulness of the hamster in understanding SARS-CoV-2 infection. First, as a small animal model, the hamster model is preferential to transgenic mouse models to investigate viral replication and transmission, and to evaluate protective effects of vaccine candidates. The hamster model also appears useful for evaluating new therapeutic agents. Hamsters are less labor-intensive and easier to handle than ferrets and show more evidence of airway disease with most strains of the virus. The anatomy and structure of the hamster lower respiratory tract differs from that of humans. The main limitation of the hamster model is that it does not reliably produce the pathological changes identified in the severe human cases of COVID-19 (ie, diffuse alveolar damage including damage to the alveolar-capillary interface due to type I pneumocyte damage, hyaline membranes, and subsequent fibrosis, or vascular thrombosis), unless hamsters of an older age group (>10 months) are used.^45^ However, in the hamster model there appears to be discordance between viral antigen immunolabeling and tissue damage in the early stage of the disease, especially after day 7 when there is pulmonary parenchymal disease but no demonstrable antigen, and so the mechanisms by which the virus infection leads to pulmonary or vascular disease are difficult to extrapolate to the human situation. For this scenario the mouse model appears better suited, and there are also more appropriate reagents available.

In summary, the 2 commonly used laboratory animals for SARS-CoV-2 infection and tropism provide useful but distinct and complimentary tools for the study of this novel coronavirus. Both are less expensive and easier to handle than ferrets, but show different disease responses. The hamster develops nonlethal infection of the conducting airways and upper respiratory tract, and thus is useful for investigation of transmission, vaccination studies, and intervention with therapeutic agents. In contrast, the K18-ACE2 transgenic mouse has no infection of the conducting airways but develops significant pulmonary disease, more analogous to that reported in human patients with severe clinical COVID-19; however, the lethality attributed to neuro-invasion including involvement of the respiratory center of the brain confounds the value of the K18-ACE2 transgenic mouse model for understanding transmission, pulmonary pathophysiology during late states of disease, and efficacy of T cell responses to vaccination and therapeutics block viral entry into permissive cells.

## Supplemental Material

Supplemental Material, sj-pdf-1-vet-10.1177_03009858211043084 - Cellular tropism of SARS-CoV-2 in the respiratory tract of Syrian hamsters and B6.Cg-Tg(K18-ACE2)2Prlmn/J transgenic mice Click here for additional data file. Supplemental Material, sj-pdf-1-vet-10.1177_03009858211043084 for Cellular tropism of SARS-CoV-2 in the respiratory tract of Syrian hamsters and B6.Cg-Tg(K18-ACE2)2Prlmn/J transgenic mice by Hui-Ling Yen, Sophie Valkenburg, Sin Fun Sia, Ka Tim Choy, J. S. Malik Peiris, Karen H. M. Wong, Nicholas Crossland, Florian Douam and John M. Nicholls in Veterinary Pathology

## References

[1] Allnoch L Beythien G Leitzen E , et al. Vascular inflammation is associated with loss of aquaporin 1 expression on endothelial cells and increased fluid leakage in SARS-CoV-2 infected golden Syrian hamsters. Viruses. 2021;13(4):639. 33918079 10.3390/v13040639 PMC8069375

[2] Atyeo C Slein MD Fischinger S , et al. Dissecting strategies to tune the therapeutic potential of SARS-CoV-2-specific monoclonal antibody CR3022. JCI Insight. 2021;6(1):e143129. 10.1172/jci.insight.143129 PMC7821590 33427208

[3] Baas T Roberts A Teal TH , et al. Genomic analysis reveals age-dependent innate immune responses to severe acute respiratory syndrome coronavirus. J Virol. 2008;82(19):9465–9476. 18632870 10.1128/JVI.00489-08 PMC2546950

[4] Basil MC Katzen J Engler AE , et al. The cellular and physiological basis for lung repair and regeneration: past, present, and future. Cell Stem Cell. 2020;26(4):482–502. 32243808 10.1016/j.stem.2020.03.009 PMC7128675

[5] Becci PJ McDowell EM Trump BF . The respiratory epithelium. II. Hamster trachea, bronchus, and bronchioles. J Natl Cancer Inst. 1978;61(2):551–561. 355650

[6] Buthala DA Soret MG . Parainfluenza type 3 virus infection in hamsters: virologic, serologic, and pathologic studies. J Infect Dis. 1964;114:226–234. 14183394 10.1093/infdis/114.3.226

[7] Calabrese F Pezzuto F Fortarezza F , et al. Pulmonary pathology and COVID-19: lessons from autopsy. The experience of European pulmonary pathologists. Virchows Arch. 2020;477(3):359–372. 32642842 10.1007/s00428-020-02886-6 PMC7343579

[8] Carossino M Montanaro P O’Connell A , et al. Fatal neuroinvasion of SARS-CoV-2 in K18-hACE2 mice is partially dependent on hACE2 expression. bioRxiv. Published online January 15, 2021. doi:10.1101/2021.01.13.425144

[9] Cleary SJ Magnen M Looney MR , et al. Update on animal models for COVID-19 research. Br J Pharmacol. 2020;177(24):5679–5681. 33140409 10.1111/bph.15266 PMC7707085

[10] Cleary SJ Pitchford SC Amison RT , et al. Animal models of mechanisms of SARS-CoV-2 infection and COVID-19 pathology. Br J Pharmacol. 2020;177(21):4851–4865. 32462701 10.1111/bph.15143 PMC7283621

[11] Deinhardt-Emmer S Bottcher S Haring C , et al. SARS-CoV-2 causes severe epithelial inflammation and barrier dysfunction. J Virol. 2021;95(10):e00110–e00121. 10.1128/JVI.00110-21 PMC8139673 33637603

[12] Deng Y Angelova A . Coronavirus-induced host cubic membranes and lipid-related antiviral therapies: a focus on bioactive plasmalogens. Front Cell Dev Biol. 2021;9:630242. 33791293 10.3389/fcell.2021.630242 PMC8006408

[13] do Vale B Lopes AP Fontes MDC , et al. Bats, pangolins, minks and other animals—villains or victims of SARS-CoV-2? Vet Res Commun. 2021;45(1):1–19. 33464439 10.1007/s11259-021-09787-2 PMC7813668

[14] Ehaideb SN Abdullah ML Abuyassin B , et al. Evidence of a wide gap between COVID-19 in humans and animal models: a systematic review. Crit Care. 2020;24(1):594. 33023604 10.1186/s13054-020-03304-8 PMC7537968

[15] Gruber AD Osterrieder N Bertzbach LD , et al. Standardization of reporting criteria for lung pathology in SARS-CoV-2-infected hamsters: what matters? Am J Respir Cell Mol Biol. 2020;63(6):856–859. 32897757 10.1165/rcmb.2020-0280LE PMC7790148

[16] Hoagland DA Moller R Uhl SA , et al. Leveraging the antiviral type I interferon system as a first line of defense against SARS-CoV-2 pathogenicity. Immunity. 2021;54(3):557–570. 33577760 10.1016/j.immuni.2021.01.017 PMC7846242

[17] Hobbs EC Reid TJ . Animals and SARS-CoV-2: species susceptibility and viral transmission in experimental and natural conditions, and the potential implications for community transmission. Transbound Emerg Dis. 2020;68(4):1850–1867. 33091230 10.1111/tbed.13885 PMC8359434

[18] Hui KPY Cheung MC Perera R , et al. Tropism, replication competence, and innate immune responses of the coronavirus SARS-CoV-2 in human respiratory tract and conjunctiva: an analysis in ex-vivo and in-vitro cultures. Lancet Respir Med. 2020;8(7):687–695. 32386571 10.1016/S2213-2600(20)30193-4 PMC7252187

[19] Imai M Iwatsuki-Horimoto K Hatta M , et al. Syrian hamsters as a small animal model for SARS-CoV-2 infection and countermeasure development. Proc Natl Acad Sci U S A. 2020;117(28):16587–16595. 32571934 10.1073/pnas.2009799117 PMC7368255

[20] Johansen MD Irving A Montagutelli X , et al. Animal and translational models of SARS-CoV-2 infection and COVID-19. Mucosal Immunol. 2020;13(6):877–891. 32820248 10.1038/s41385-020-00340-z PMC7439637

[21] Kaptein SJF Jacobs S Langendries L , et al. Favipiravir at high doses has potent antiviral activity in SARS-CoV-2-infected hamsters, whereas hydroxychloroquine lacks activity. Proc Natl Acad Sci U S A. 2020;117(43):26955–26965. 33037151 10.1073/pnas.2014441117 PMC7604414

[22] Kurup D Malherbe DC Wirblich C , et al. Inactivated rabies virus vectored SARS-CoV-2 vaccine prevents disease in a Syrian hamster model. PLoS Pathog. 2021;17(3):e1009383. 33765062 10.1371/journal.ppat.1009383 PMC8023494

[23] Li C Chen YX Liu FF , et al. Absence of vaccine-enhanced disease with unexpected positive protection against SARS-CoV-2 by inactivated vaccine given within three days of virus challenge in Syrian hamster model. Clin Infect Dis. 2021;73(3):e719–e734. 33515458 10.1093/cid/ciab083 PMC7929057

[24] Liu L Wang P Nair MS , et al. Potent neutralizing antibodies against multiple epitopes on SARS-CoV-2 spike. Nature. 2020;584(7821):450–456. 32698192 10.1038/s41586-020-2571-7

[25] Lu M Dravid P Zhang Y , et al. A safe and highly efficacious measles virus-based vaccine expressing SARS-CoV-2 stabilized prefusion spike. Proc Natl Acad Sci U S A. 2021;118(12):e2026153118. 33688034 10.1073/pnas.2026153118 PMC8000430

[26] MacPhail M Schickli JH Tang RS , et al. Identification of small-animal and primate models for evaluation of vaccine candidates for human metapneumovirus (hMPV) and implications for hMPV vaccine design. J Gen Virol. 2004;85(Pt 6):1655–1663. 15166450 10.1099/vir.0.79805-0

[27] Mason RJ . Thoughts on the alveolar phase of COVID-19. Am J Physiol Lung Cell Mol Physiol. 2020;319(1):L115–L120. 32493030 10.1152/ajplung.00126.2020 PMC7347958

[28] Munoz-Fontela C Dowling WE Funnell SGP , et al. Animal models for COVID-19. Nature. 2020;586:509–515. 32967005 10.1038/s41586-020-2787-6 PMC8136862

[29] Nambulli S Xiang Y Tilston-Lunel NL , et al. Inhalable Nanobody (PiN-21) prevents and treats SARS-CoV-2 infections in Syrian hamsters at ultra-low doses. Sci Adv. 2021;7(22):eabh0319. 34039613 10.1126/sciadv.abh0319 PMC8153718

[30] Nicholls JM Butany J Poon LL , et al. Time course and cellular localization of SARS-CoV nucleoprotein and RNA in lungs from fatal cases of SARS. PLoS Med. 2006;3(2):e27. 16379499 10.1371/journal.pmed.0030027 PMC1324951

[31] Nicholls JM Poon LL Lee KC , et al. Lung pathology of fatal severe acute respiratory syndrome. Lancet. 2003;361(9371):1773–1778. 12781536 10.1016/S0140-6736(03)13413-7 PMC7112492

[32] Osterrieder N Bertzbach LD Dietert K , et al. Age-dependent progression of SARS-CoV-2 infection in Syrian hamsters. Viruses. 2020;12(7):779. 10.3390/v12070779 PMC7412213 32698441

[33] Pandey K Acharya A Mohan M , et al. Animal models for SARS-CoV-2 research: a comprehensive literature review. Transbound Emerg Dis. 2020;68(4):1868–1885. 33128861 10.1111/tbed.13907 PMC8085186

[34] Plante KS Dwivedi V Plante JA , et al. Antiviral activity of oleandrin and a defined extract of Nerium oleander against SARS-CoV-2. Biomed Pharmacother. 2021;138:111457. 33721754 10.1016/j.biopha.2021.111457 PMC7927596

[35] Ray S Chiba N Yao C , et al. Rare SOX2^+^ airway progenitor cells generate krt5^+^ cells that repopulate damaged alveolar parenchyma following influenza virus infection. Stem Cell Reports. 2016;7(5):817–825. 27773701 10.1016/j.stemcr.2016.09.010 PMC5106521

[36] Rehm S Takahashi M Ward JM , et al. Immunohistochemical demonstration of Clara cell antigen in lung tumors of bronchiolar origin induced by N-nitrosodiethylamine in Syrian golden hamsters. Am J Pathol. 1989;134(1):79–87. 2464284 PMC1879554

[37] Roberts A Lamirande EW Vogel L , et al. Immunogenicity and protective efficacy in mice and hamsters of a beta-propiolactone inactivated whole virus SARS-CoV vaccine. Viral Immunol. 2010;23(5):509–519. 20883165 10.1089/vim.2010.0028 PMC2967819

[38] Roberts A Lamirande EW Vogel L , et al. Animal models and vaccines for SARS-CoV infection. Virus Res. 2008;133(1):20–32. 17499378 10.1016/j.virusres.2007.03.025 PMC2323511

[39] Roberts A Vogel L Guarner J , et al. Severe acute respiratory syndrome coronavirus infection of Golden Syrian hamsters. J Virol. 2005;79(1):503–511. 15596843 10.1128/JVI.79.1.503-511.2005 PMC538722

[40] Rosa RB Dantas WM do Nascimento JCF , et al. In vitro and in vivo models for studying SARS-CoV-2, the etiological agent responsible for COVID-19 pandemic. Viruses. 2021;13(3):379. 33673614 10.3390/v13030379 PMC7997194

[41] Rosenke K Jarvis MA Feldmann F , et al. Hydroxychloroquine prophylaxis and treatment is ineffective in macaque and hamster SARS-CoV-2 disease models. JCI Insight. 2020;5(23):e143174. 10.1172/jci.insight.143174 PMC7714406 33090972

[42] Sanchez-Felipe L Vercruysse T Sharma S , et al. A single-dose live-attenuated YF17D-vectored SARS-CoV-2 vaccine candidate. Nature. 2021;590(7845):320–325. 33260195 10.1038/s41586-020-3035-9

[43] Sanclemente-Alaman I Moreno-Jimenez L Benito-Martin MS , et al. Experimental models for the study of central nervous system infection by SARS-CoV-2. Front Immunol. 2020;11:2163. 32983181 10.3389/fimmu.2020.02163 PMC7485091

[44] Schafer A Muecksch F Lorenzi JCC , et al. Antibody potency, effector function, and combinations in protection and therapy for SARS-CoV-2 infection in vivo. J Exp Med. 2021;218(3):e20201993. 33211088 10.1084/jem.20201993 PMC7673958

[45] Selvaraj P Lien CZ Liu S , et al. SARS-CoV-2 infection induces protective immunity and limits transmission in Syrian hamsters. Life Sci Alliance. 2021;4(4):e20200 0886. 10.26508/lsa.202000886 PMC7893819 33574037

[46] Sia SF Yan LM Chin AWH , et al. Pathogenesis and transmission of SARS-CoV-2 in golden hamsters. Nature. 2020;583(7818):834–838. 32408338 10.1038/s41586-020-2342-5 PMC7394720

[47] Sridhar S Nicholls J . Pathophysiology of infection with SARS-CoV-2—what is known and what remains a mystery. Respirology. 2021;26(7):652–665. 34041821 10.1111/resp.14091 PMC8242464

[48] Sun W McCroskery S Liu WC , et al. A Newcastle disease virus (NDV) expressing a membrane-anchored spike as a cost-effective inactivated SARS-CoV-2 vaccine. Vaccines (Basel). 2020;8(4):771. 10.3390/vaccines8040771 PMC7766959 33348607

[49] Suprewicz L Swoger M Gupta S , et al. Vimentin binds to SARS-CoV-2 spike protein and antibodies targeting extracellular vimentin block in vitro uptake of SARS-CoV-2 virus-like particles. bioRxiv. Published online January 8, 2021. doi:10.1101/2021.01.08.425793

[50] Vanderheiden A Ralfs P Chirkova T , et al. Type I and type III interferons restrict SARS-CoV-2 infection of human airway epithelial cultures. J Virol. 2020;94(19):e00985–e00920. 10.1128/JVI.00985-20 PMC7495371 32699094

[51] Ye G Gallant JP Massey C , et al. The development of a novel nanobody therapeutic for SARS-CoV-2. bioRxiv. Published online November 17, 2020. doi:10.1101/2020.11.17.386532

[52] Ye ZW Yuan S Chan JFW , et al. Beneficial effect of combinational methylprednisolone and remdesivir in hamster model of SARS-CoV-2 infection. Emerg Microbes Infect. 2021;10(1):291–304. 33538646 10.1080/22221751.2021.1885998 PMC7919885

[53] Younes S Younes N Shurrab F , et al. Severe acute respiratory syndrome coronavirus-2 natural animal reservoirs and experimental models: systematic review. Rev Med Virol. 2020;32(4):e2196. 10.1002/rmv.2196 PMC7744864 33206434

[54] Yuan S Wang R Chan JFW , et al. Metallodrug ranitidine bismuth citrate suppresses SARS-CoV-2 replication and relieves virus-associated pneumonia in Syrian hamsters. Nat Microbiol. 2020;5(11):1439–1448. 33028965 10.1038/s41564-020-00802-x

