# Supplementary material for: Cellular tropism of SARS-CoV-2 in the respiratory tract of Syrian hamsters and B6.Cg-Tg(K18-ACE2)2Prlmn/J transgenic mice
Source: Vet Pathol. 2021 Sep 1;59(4):639–47. doi: 10.1177/03009858211043084 (PMC8721337; doi:10.1177/03009858211043084)
Supplement: Supplemental Material, sj-pdf-1-vet-10.1177_03009858211043084 - Cellular tropism of SARS-CoV-2 in the respiratory tract of Syrian hamsters and B6.Cg-Tg(K18-ACE2)2Prlmn/J transgenic mice [file sj-pdf-1-vet-10.1177_03009858211043084.pdf]

Yen et al. Cellular tropism of SARS-CoV-2 in the respiratory tract of Syrian Hamsters and B6.Cg-Tg(K18-ACE2)2Prlmn/J transgenic mice.

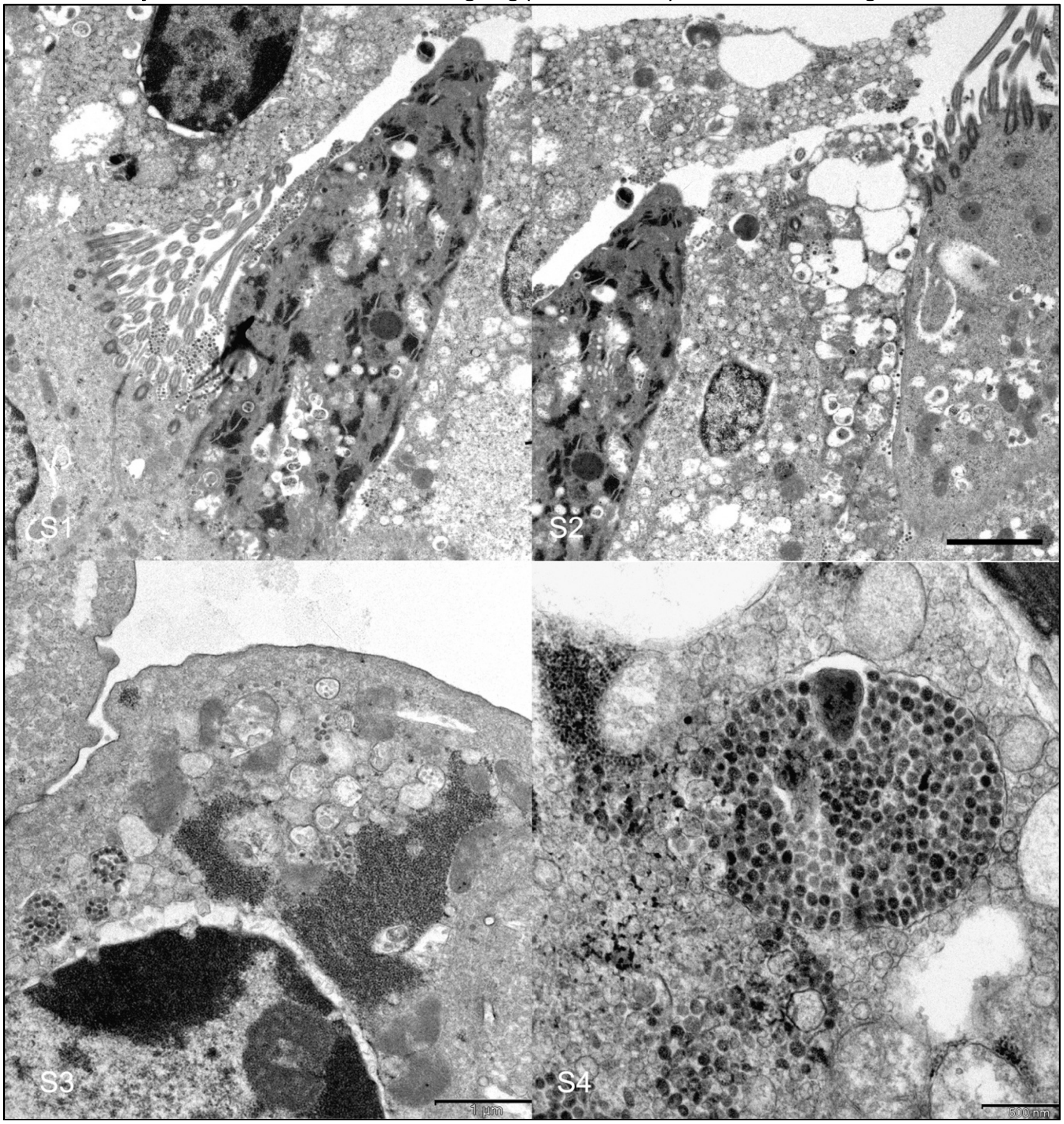

Supplemental Figure S1 Bronchiolar epithelium, hamster. Transmission electron microscopy (TEM). There is marked cytoplasmic swelling and vacuolation of a non-ciliated cell. Aggregates of viral particles are present in secretory vesicles.

Supplemental Figure S2 Bronchiolar epithelium, hamster. TEM. There is marked cytoplasmic swelling and vacuolation of a non-ciliated cell. Aggregates of viral particles are present in secretory vesicles, on the surface, and in the intercellular space between the ciliated cell and the non-ciliated cells. In contrast to the non-ciliated cell, the cell membrane of the ciliated cell is relatively intact.

Supplemental Figure S3: Bronchiolar epithelium, hamster.TEM. A non-ciliated cell contains large membrane-bound aggregate of viral particles.

Supplemental Figure S4. Bronchiolar epithelium, hamster. TEM. A ciliated epithelial cell contains large membrane-bound aggregates of viral particles on the basal aspect of the cell.

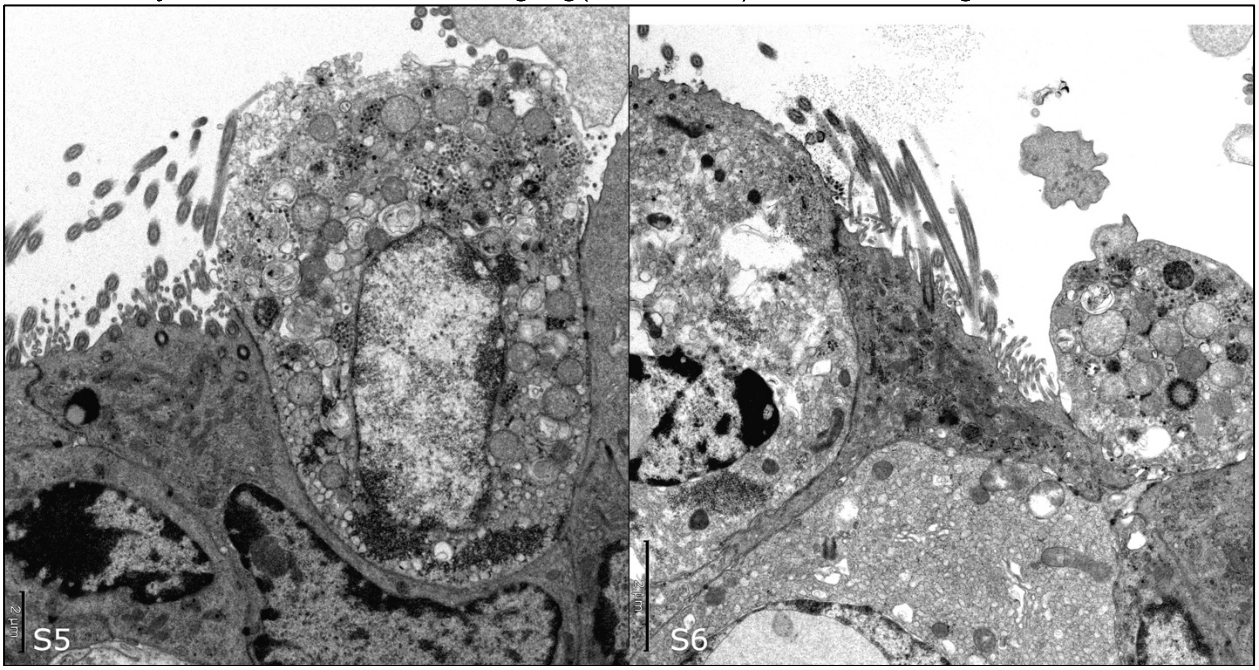

Supplemental Figure S5. Bronchiolar epithelium, hamster. TEM, A non-ciliated cell has cell swelling, membrane fragmentation, and cytoplasmic membrane-bound aggregates of large numbers of viral particles. In contrast, the adjacent ciliated cell shows minimal cytopathic changes.

Supplemental Figure S6. Bronchiolar epithelium, hamster. TEM. A ciliated cell has small clusters of extracellular viral particles on the cell surface. The non-ciliated cell fragment contains large numbers of membrane- and non-membrane-bound viral particles, including one aggregate of closely packed particles.
